# MYC Deregulation in Primary Human Cancers

**DOI:** 10.3390/genes8060151

**Published:** 2017-05-25

**Authors:** Manpreet Kalkat, Jason De Melo, Katherine Ashley Hickman, Corey Lourenco, Cornelia Redel, Diana Resetca, Aaliya Tamachi, William B. Tu, Linda Z. Penn

**Affiliations:** 1 Department of Medical Biophysics, University of Toronto, Toronto, ON M5G 1L7, Canada; manpreet.kalkat@mail.utoronto.ca (M.K.); ashley.hickman@mail.utoronto.ca (K.A.H.); corey.lourenco@mail.utoronto.ca (C.L.); cornelia.redel@mail.utoronto.ca (C.R.); diana.resetca@mail.utoronto.ca (D.R.); william.tu@mail.utoronto.ca (W.B.T.); 2 Princess Margaret Cancer Centre, University Health Network, Toronto, ON M5G 2M9, Canada; jason.demelo@uhnresearch.ca (J.D.M.); aaliya.tamachi@utoronto.ca (A.T.); 3 Sunnybrook Research Institute, Toronto, ON M4N 3M5, Canada

**Keywords:** MYC, deregulation, cancer, gene amplification, translocation, enhancers, cell signaling

## Abstract

MYC regulates a complex biological program by transcriptionally activating and repressing its numerous target genes. As such, MYC is a master regulator of many processes, including cell cycle entry, ribosome biogenesis, and metabolism. In cancer, the activity of the MYC transcriptional network is frequently deregulated, contributing to the initiation and maintenance of disease. Deregulation often leads to constitutive overexpression of MYC, which can be achieved through gross genetic abnormalities, including copy number alterations, chromosomal translocations, increased enhancer activity, or through aberrant signal transduction leading to increased *MYC* transcription or increased MYC mRNA and protein stability. Herein, we summarize the frequency and modes of MYC deregulation and describe both well-established and more recent findings in a variety of cancer types. Notably, these studies have highlighted that with an increased appreciation for the basic mechanisms deregulating MYC in cancer, new therapeutic vulnerabilities can be discovered and potentially exploited for the inhibition of this potent oncogene in cancer.

## 1. Introduction

MYC (c-MYC) regulates the expression of many target genes that together coordinate a complex biological response to coordinate cell growth and proliferation. MYC achieves this as a sequence specific transcription factor, that together with its partner protein MAX [1,2], regulates the activation [1] and repression [3,4] of its many target genes necessary for cell cycle progression, ribosome biogenesis, and biomass accumulation [5,6,7]. More recently, MYC has also been implicated in the regulation of transcriptional pause release of RNA Polymerase II [8,9,10] and enhanced capping of nascent mRNA transcripts [11,12], leading to a pervasive effect on transcript abundance [13].

MYC RNA and protein expression is normally tightly regulated and increases in response to discrete biological scenarios requiring high MYC levels, such as cellular proliferation in response to mitogenic signaling [7,14,15,16]. However, in cancer, MYC expression and/or activity are frequently co-opted by the tumor, resulting in elevated MYC RNA and protein expression; accordingly, MYC has been identified as one of the most commonly deregulated oncogenes in a wide variety of cancer types [17,18]. This aberrant expression, or deregulation, of MYC and the transforming MYC family members (N-MYC and L-MYC) can take many different forms, reflecting the complexity of MYC biological regulation. Broadly, MYC deregulation can be classified as alterations that result in MYC RNA or protein products that are unresponsive to negative regulatory stimuli, resulting in sustained MYC expression, or result in elevated basal levels of MYC expression. Herein, we summarize recent findings regarding the variety of mechanisms by which MYC can be deregulated in cancer, contributing to its potent role in both the initiation and maintenance of the tumorigenic state.

## 2. MYC Amplification

**Figure 1:**
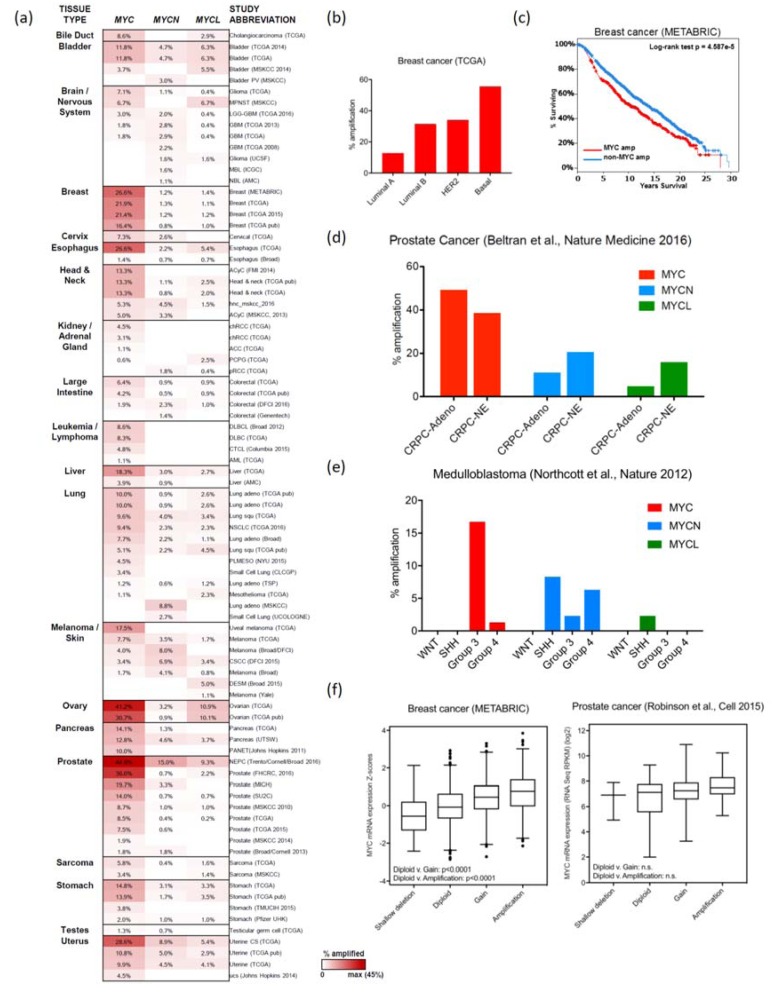
MYC, *MYCN*, *MYCL* amplifications in cancer. (**a**) A heatmap depicting the frequency of copy number alterations (CNAs) in *MYC*, *MYCN*, and *MYCL* loci across multiple cancers grouped based on tissue of origin (data mined from cbioportal.org [34,35]). The source of the genomic data is indicated in parentheses. (**b**) *MYC* amplification identified in four molecular subtypes of breast cancer [30]. (**c**) Long-term survival analysis of 2051 breast cancer patients (over 30 years) with *MYC*-amplified and non-*MYC*-amplified cancers (data accessed through cbioportal.org and METABRIC [29]). (**d**) Amplification of *MYC*, *MYCN*, and *MYCL* identified in two molecular subtypes of prostate cancer, castration-resistant prostate cancer (CRPC) adenocarcinoma and neuroendocrine-CRPC [36]. (**e**) Amplification of *MYC*, *MYCN*, and *MYCL* identified in four molecular subtypes of medulloblastoma [37]. (**f**) Correlation of MYC mRNA expression with the type of copy number alteration CNA (deletion, ploidy, gain, or amplification) in breast cancer (left, METABRIC) and prostate cancer [38] (right).

Gene amplification of *MYC* is the most commonly observed marker of MYC deregulation in cancer. Gene duplication, taking place through genome doubling or tandem duplications [19], is the underlying mechanism for copy-number alterations (CNAs) in various oncogenes. Evidence of *MYC* amplification was first identified in the human leukemia cell line HL60 in 1982 [20]. Soon after, amplifications of *MYCN* and *MYCL* were discovered in cell lines and tumors of neuroblastoma and small cell lung cancer, respectively [21,22], with small cell lung cancer being the first cancer found to have amplifications of all three transforming *MYC* family members [22,23,24]. In the past decade, the multitude of genome-wide sequencing studies of primary tumors, enabled by technological advances in next-generation sequencing and data analysis [25], further established *MYC* as one of the most frequently amplified genes across human cancers and revealed new cancer types that harbor *MYC*, *MYCN*, and *MYCL* amplifications (Summarized in Figure 1a). Comprehensive global genome-sequencing projects on multitudes of cancers, like The Cancer Genome Atlas (TCGA) [26] and International Cancer Genome Consortium (ICGC) [27] facilitated the detection of previously unknown somatic mutations and CNAs and linked these to clinical data. Pan-cancer analyses of at least 12 cancer types estimated the frequency of *MYC* amplification at approximately 14% [17,18,28]. One such study categorized cancers into two classes, one dominated by somatic mutations and the other by CNAs. In the latter class, comprised primarily of ovarian, breast, and squamous cell lung cancers, *MYC* amplification was the top CNA [18].

### 2.1. Breast Cancer

Two of the largest genomic studies of breast cancers, analyzing a collection of more than 3000 breast tumors was performed by the Molecular Taxonomy of Breast Cancer International Consortium (METABRIC) and TCGA. These studies identified *MYC* amplifications in 26.6% and 21.9% of samples analysed, respectively (Figure 1a) [29,30]. Breast cancer can be divided into distinct subtypes based on histological and molecular classifications that have prognostic and therapeutic values. *MYC* amplification and MYC pathway activation are hallmark features of the basal subtype (55.6% with *MYC* amplification), a subtype associated with aggressive disease and poor prognosis, and lacking targeted therapeutic options [18]. *MYC* amplification is also present in a substantial portion of other subtypes representing receptor-positive disease: HER2-positive (34.1%), luminal B (31.5%), and luminal A (12.8%) (Figure 1b) [18]. *MYC* amplification is also associated with poor overall survival and poor recurrence-free rates, with *MYC* copy number gains increasing post-treatment in some instances (Figure 1c) [31]. It is important to note that *MYC* amplification is not the only mechanism to deregulate MYC in breast cancer (see Section 5.4).

### 2.2. Ovarian and Endometrial Cancers

Ovarian and endometrial cancers are two common cancers in women, and across both cancers, patients with advanced stage, high-grade subtypes have a high incidence of mortality. TCGA analyses of ovarian carcinomas (557 samples) and endometrial carcinomas (373 samples) showed that *MYC* is amplified in 30.7% and 10.8% of tumors, respectively [32,33]. In ovarian cancer, *MYC* amplification is correlated with inactivation in the breast and ovarian cancer susceptibility protein (BRCA) and retinoblastoma-associated protein (RB1) pathways [32]; in endometrial cancer, *MYC* amplification is associated with low estrogen receptor (ER)/FOXA1 activity and *TP53* mutations [33]. The high-grade subtype of both ovarian and endometrial cancers harbors a high frequency of *MYC* amplification, along with *TP53*, *BRCA1/2* mutations, and *RB1* loss [30,33].

### 2.3. Colorectal Cancer

Colorectal cancer is a leading cause of cancer mortality in the world, and can be subdivided into four consensus molecular subtypes (CMS1-4), as defined by transcriptomics with different genetic and biological features [39]. TCGA analysis (220 samples) and a larger meta-analysis (503 samples) identified the frequency of MYC amplification at 6% overall, while frequencies in the different subtypes ranged from 5% to 11% [39,40]. The MYC transcriptional program is specifically enriched in the epithelial CMS2 subtype, with activated WNT pathway signaling [39,40,41], which has been shown to transcriptionally activate *MYC* expression [42] (also further discussed in Section 3.2 and Section 5.5). Furthermore, frequent inactivating mutations of the E3 ubiquitin ligase *FBXW7* and transcriptional regulators *SMAD* and *ARID1A* may result in increased protein stability and mRNA transcription, respectively [40,43,44,45,46,47].

### 2.4. Prostate Cancer

Prostate cancer is one of the most common adult malignancies, with a subset of men progressing to the development of aggressive metastatic disease. In a TCGA study of 333 primary prostate carcinomas, *MYC* focal amplifications were reported in 8% of the tumors [48]. A second study of 277 non-indolent localized prostate cancers identified recurrent CNAs, including amplification of *MYC*, and deletion of *PTEN*, *TP53*, and *NKX3-1* [49]. Overall, these studies indicated evidence of MYC family involvement in prostate cancer (Figure 1a,d). Patients with metastatic castration-resistant prostate cancer (CRPC) treated androgen-directed therapies, including enzalutamide [50] and abiraterone acetate [51], eventually develop resistance characterized by low or absent androgen receptor (AR) expression and a neuroendocrine (CRPC-NE) phenotype. In addition to the loss of RB1 (70% of CRPC-NE) and mutations or deletions of *TP53* (67% of CRPC-NE), somatic allele-specific CNAs of *MYC* and *MYCN* were evident in 45% and 15% of CRPC-NE, respectively. This suggests a strong evolutionary divergence in the development of neuroendocrine prostate cancer from the prostate adenocarcinoma, and hints at differential importance of *MYC* amplification in these subtypes [36]. Additional mechanisms of MYC deregulation in prostate cancer are further discussed in Section 3.2.

### 2.5. Lung Cancer

Lung cancer can be characterized by two histopathological classes: small-cell lung cancer (SCLC) and non-small-cell lung cancer (NSCLC); the latter being the predominant class. The SCLC cell of origin has been proposed to be of neuroendocrine character, while NSCLC comprises squamous cell carcinoma (SCC), which originates from the proximal airway and adenocarcinoma that arises from distal regions and classifies into multiple subtypes [52]. A TCGA study of 178 SCC lung patient samples provided insights into the genomic and epigenomic status of these tumors, with an average of 323 CNAs, 360 exonic mutations, and 165 genomic rearrangements per tumor reported, including *MYC* and *MYCL* amplifications observed at a frequency of 5% and 4%, respectively [53].

In a recent study, 183 NSCLC adenocarcinomas were profiled with whole-exome/genome sequencing [54]. Among the 25 genetic alterations identified, *MYC* amplification was observed in 31% of cases. In another comprehensive analysis of lung adenocarcinoma, somatic CNAs exhibited a very similar profile with significant *MYC* amplification [55] (Figure 1a). Recurrent loss-of-function mutations in the *MGA* gene, which encodes a MAX interactor, are observed in 8% of lung adenocarcinoma specimens and appear to be mutually exclusive with *MYC* amplification [55]. In another study that comparatively analyzed 660 lung adenocarcinomas and 484 SCC cases *MYC* amplification was observed in both adenocarcinoma and SCC tumors, with *MYCL* significantly more amplified in lung adenocarcinoma. Additional mechanisms of MYC deregulation in lung cancer are further discussed in Section 2.9 and Section 3.2.

### 2.6. Pancreatic Cancer

In a study of over 500 pancreatic ductal adenocarcinoma (PDAC) samples, *MYC* amplification was observed in 14% of PDAC and was enriched in the aggressive adenosquamous histological subtype [56,57]. *MYC* copy number was also an independent marker of poor outcome, with an overall survival of less than 10 months in the high copy number cohort [57].

### 2.7. Renal Clear Cell Carcinoma and Adrenal Cell Carcinoma

Renal cell carcinoma (RCC) is the most common type of kidney cancer, with clear cell RCC (ccRCC) representing approximately 75% of all RCCs. In a study by Sato et al., more than 100 ccRCC cases were analyzed by whole-genome, whole-exome, and RNA sequencing, as well as array-based gene expression, copy number and methylation analyses [58], finding significant focal alterations at 20 loci, including copy number gains in *MYC* in 23% of the cases. TCGA analysis investigated more than 400 tumors and observed amplifications in *MYC* in 15% of cases [59].

### 2.8. MYC CNAs in Pediatric Cancers

#### 2.8.1. Medulloblastoma

Medulloblastoma is a paediatric malignant brain tumor that can be classified into four distinct subtypes as the result of transcriptional profiling: two that are associated with deregulation in the WNT/β-catenin and sonic hedgehog (SHH) signaling pathways, and two less well-characterized subtypes, termed Groups 3 and 4 [60]. MYC and its family members have been implicated in all four subtypes (Figure 1e, see also Section 5.5), with *MYC* amplification and elevated MYC mRNA expression predominantly observed in Group 3 [61]. In a genomic study of 827 tumors, 16.7% of Group 3 tumors were *MYC*-amplified [37]. Three other studies showed 3–17.6% of cases with *MYC* amplifications in Group 3 [61,62,63]. In this subtype, MYC expression may also be elevated through additional non-coding mechanisms: (1) *PVT1*-*MYC* fusions exist in 60% of *MYC*-amplified cases, and the long non-coding RNA *PVT1* creates a positive feedback loop to maintain high MYC expression [37]; (2) the *MYC* amplicon contains a medulloblastoma-specific enhancer (high histone H3K27 acetylation clusters) to enforce high MYC expression (see also Section 3.2) [64].

*MYC* and *MYCN* are target genes of the WNT/β-catenin and SHH pathways, respectively, with elevated mRNA expression seen in these two subtypes [61]. The SHH subtype also harbors *MYCN*-amplified (7.7–16.7%) and *MYCL*-amplified tumors (2.3%) [37,61,62,63], although the biological role of L-MYC in medulloblastoma is unknown (Figure 1e). Group 4 also has 5–10.5% of cases with *MYCN* amplification [37,61,62,63]. This, along with N-MYC-driven mouse models that give rise to Group 4-like medulloblastoma [65,66], implicates N-MYC in the oncogenesis of this subtype.

In a genomic study comparing primary and metastatic tumor samples, *MYC* amplification was present in matched primary tumors and metastases, whereas *MYCN* amplification was only present in primary tumors but not their matched metastases [67]. This suggests that MYC and N-MYC may be involved in different stages of the oncogenic process. Increased frequencies of *MYC* and *MYCN* amplifications also emerged in tumors of relapsed patients compared to at diagnosis [68,69]. The aggressive nature of these relapsed tumors may be driven by the combined acquisition of *MYC*/*MYCN* amplifications and *TP53* mutations [68]. The diversity of MYC family deregulation in medulloblastoma illustrates that, while MYC family members can all drive oncogenesis, they may have distinct roles in giving rise to cancers of different molecular and clinical phenotypes.

#### 2.8.2. Neuroblastoma

Neuroblastoma is a childhood malignancy that afflicts the developing sympathetic nervous system and accounts for up to 10% of all childhood cancers, with 80% of neuroblastomas occurring before the age of five. Early work identified *MYCN* amplification in approximately 40% of patient cases, correlating with advanced disease (stage III or IV) and poor prognosis [70]. The majority of neuroblastoma patients can be classified into three distinct genomic types [71]: type A tumors with only numerical changes of whole chromosomes, but without any detectable structural rearrangement; type B tumors characterized by the presence of only partial chromosome imbalances (excluding *MYCN* amplification) in the absence of any numerical chromosomal aberrations; and type C tumors that harbor *MYCN* amplification without numerical chromosomal aberrations. Two additional genomic types, D and E, account for mixed profiles with respect to their segmental or numerical aberrations and *MYCN* amplification. Recently, Westermann et al. examined the relationship between *MYC* and *MYCN* amplification in neuroblastoma, which are known to transcriptionally repress of one another [72]. They found that MYC expression in *MYCN* amplified neuroblastoma is not generally observed, and when MYC is over-expressed it is dominant over N-MYC expression [72].

Recently, a number of whole-genome and whole-exome sequencing studies have broadened our understanding of the role *MYCN* amplification plays in this cancer. The Therapeutically Applicable Research to Generate Effective Treatments (TARGET) initiative analyzed specimens from 240 cases by a combination of whole-exome and whole-genome sequencing [73], where amplification of the *MYCN* oncogene was observed in 32% of the cases (confirmed by fluorescence in situ hybridization) and a recurrent *MYCN* mutation was observed in 1.7% of the cases. Another study, consisting of a whole-genome sequencing data of 87 neuroblastoma specimens identified chromothripsis, a local shredding of chromosomes, in 18% of late stage neuroblastomas associated with poor outcomes [74]. These chromothripsis-related structural aberrations were associated with amplification of *MYCN*, and in one tumor, resulted in *MYC* amplification and overexpression [74].

A study examining 217 neuroblastoma tumors by whole-genome sequencing, including 75 high-risk tumors, found that most high-risk tumors harbored either *MYCN* amplification, *TERT* rearrangements or *ATRX* mutations, all of which can contribute to telomere lengthening and could be useful in the molecular classification of high-risk neuroblastoma [75]. *MYCN* is a known transcriptional activator of TERT, yet *MYCN* amplifications were mutually exclusive with *TERT* rearrangements, as well as *ATRX* alterations, a gene associated with alternate lengthening of telomeres, suggesting that these alterations may share a similar role in neuroblastoma [75].

### 2.9. MAX Alterations

MYC requires its basic helix-loop-helix leucine zipper (bHLHLZ) dimerization partner MAX for its transcriptional and oncogenic activities [1,2,4,76] however, a rat pheochromocytoma cell line (PC-12) does not express MAX mRNA or protein, yet retains a transcriptionally functional MYC protein [77,78]. A number of genomic sequencing studies have identified inactivating *MAX* mutations and deletions in human pheochromocytomas [79,80], small-cell lung tumors [81], and oligodendroglial tumors [82]. In the latter two cancer types, *MAX* alterations appear to be mutually exclusive to amplifications of *MYC* and *MYC* family members [81,82]. The mechanisms by which MYC functions without its dimerization partner MAX, particularly in the context of cancer, remain to be elucidated, but does suggest that there are additional means of activating the MYC transcriptional program.

### 2.10. Considerations for MYC Amplification

*MYC* amplification is observed in virtually all cancer types. The abundance of recent genomic sequencing studies has indicated that *MYC* amplification can often define specific molecular subtypes, and is frequently associated with aggressive disease, metastatic potential, therapeutic resistance and poor patient outcomes (Figure 1c). The sequencing of extrachromosomal DNA (ecDNA) in multiple cancer types identified a significantly higher copy number of oncogenes, including *MYC*, in ecDNA compared to chromosomal DNA, suggesting additional means for tumors to gain copies of *MYC* that contribute to tumor adaptability and heterogeneity [83]. As another layer of complexity, amplification of each of the three oncogenic *MYC* family members can be present in cancer, yet each may give rise to distinct biological phenotypes.

While *MYC* amplification is an overt sign of MYC deregulation, the mechanism by which this CNA drives oncogenesis is not entirely clear. The simplest explanation is that *MYC* amplification leads to increased MYC mRNA and protein levels, and this elevated level is sufficient to elicit proliferative and oncogenic transcriptional programs. *MYC* amplification, however, is not always correlated with increased MYC mRNA or protein levels [84,85,86] (Figure 1f), suggesting: (1) MYC levels can be elevated through transcriptional (e.g., Wingless (WNT) and NOTCH signaling) [42,87] and post-transcriptional (e.g., FBXW7 ubiquitin ligase and RAS/MAPK pathway) [43,44,88] mechanisms; (2) *MYC* amplification may require cooperating events to deregulate MYC levels or activity. For example, the long non-coding RNA gene, *PVT1*, which resides in the 8q24.21 *MYC* amplicon, is necessary for high MYC expression, mediated by a positive feedback loop [37,89]. In *MYC*-amplified tumors, the presence of *TP53* mutations, a frequently co-occurring event with *MYC* amplification in many tumor types, correlates with higher *MYC* mRNA expression compared to *TP53* wild-type tumors [90]. One hypothesis is that *MYC* amplification and elevated MYC expression may have contributed to the initiation of pre-cancerous lesions [91], but those clones that progress have lower levels of deregulated MYC expression [92]. More research in this area is required to fully appreciate the contribution of *MYC* amplification to tumorigenesis.

## 3. Enhancer Activity

The tightly coordinated control of MYC expression in response to extracellular and intracellular signaling is accomplished via cell-type specific enhancer sequences surrounding the *MYC* locus. In cancer, constitutively high levels of MYC expression can be achieved by translocations rendering *MYC* under the control of highly active lineage-specific enhancers, such as in Burkitt Lymphoma (BL), or through the acquisition of increased enhancer activity affecting MYC transcription from the endogenous locus. These modes of MYC deregulation will be discussed below.

### 3.1. Translocations

**Figure 2:**
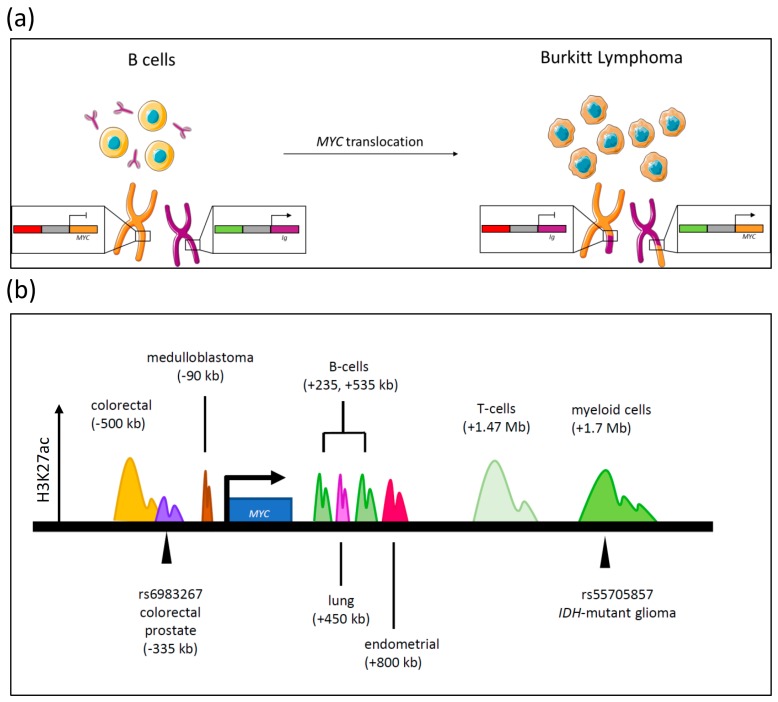
Enhancer alterations deregulating *MYC* transcription. (**a**) In B-cells, the immunoglobulin (*Ig*) gene locus (left) is controlled by an upstream enhancer that ensures constitutive high expression levels of antibodies. In Burkitt Lymphoma, the *Ig* and *MYC* loci translocate rendering *MYC* under the influence of a highly active enhancer (right). Translocation can be visualized on a chromosomal level when comparing the length of sister chromatids in anaphase spread assays. (**b**) Deregulation of *MYC* by regulatory elements at the *MYC* locus. Lineage-specific super-enhancers have been mapped to the gene desert area surround *MYC* at the 8q24.21 locus, as indicated by colored cartoon peaks representing H3K27 acetylation. Two single nucleotide polymorphisms (SNPs) have been linked to altered *MYC* transcription: rs6983267, located 335 kb upstream of the *MYC* locus, and rs55705857, located within an enhancer 1.7 Mb downstream of *MYC*.

One of the first means of MYC deregulation to be discovered were *MYC* translocations, the defining genetic feature of BL, a subtype of Non-Hodgkin B-cell lymphoma [93,94,95,96,97]. In the 1980s, three independent research groups identified a translocation of the *MYC* gene locus from its endogenous location on chromosome 8 [98,99,100,101] (Figure 2a). While a common feature of these translocations was the placement of *MYC* under the control of an unrelated distal enhancer, further investigation revealed that 70–80% of BL patients possess a t(8;14)(q24;q32) translocation rendering *MYC* under the control of the immunoglobulin (Ig) heavy chain enhancer, whereas 10–15% of patients acquire a t(2;8)(p12;q24) or t(8;22)(q24;q11) karyotype, related to translocation to the κ and λ light chain enhancers, respectively [97,99].

Another B-cell malignancy, displaying equally high MYC protein levels, is multiple myeloma (MM) [102]. MM is a highly diverse disease that displays a large range of non-random translocation events into the *Ig* heavy and light chain loci, with five non-*MYC* gene loci also being prevalent [103,104,105]. Nevertheless, 40 out of 43 independent myeloma cell lines were shown to possess *MYC* translocation with a majority involving an *Ig* locus [106]. However, the breakpoints of these rearrangements did not occur proximal to within the *Ig* switch regions [106], leading to the assumption that primary translocations increase genomic instability in these cells, thereby rendering them more vulnerable for eventual *MYC* translocation in the transition of a pre-malignant state to MM [106]. Consistently, MYC deregulation was observed in 67% of MM patients [102,107].

### 3.2. Activated Enhancer Activity

Translocations observed in B-cell malignancies have established the paradigm of altered enhancer activity in *MYC* deregulation. More recently, altered enhancer activity has also been identified as a mechanism contributing to deregulation of *MYC* at the endogenous genomic locus. The *MYC* gene exists within a “gene desert” region on chromosome 8q24, located within a 2 Mb stretch of the genome that is notable for the relative infrequency of protein-coding sequences [108,109]. Stretches of this region have the capacity to act as *MYC*-specific enhancers to fine-tune *MYC* expression. Complicating this scenario further, activation of endogenous enhancers through gene duplication or epigenetic mechanisms has been demonstrated to be another means to deregulate MYC expression from the endogenous locus [110,111,112].

#### 3.2.1. Single Nucleotide Polymorphisms and *MYC* Transcription

Genome-wide association studies (GWAS) have indicated that single nucleotide polymorphisms (SNPs) frequently map to non-coding regulatory locations in the genome. This includes enhancer regions, and in some cases SNPs have been implicated in altering enhancer activity [113,114]. Several cancer-associated SNPs have been mapped to the 8q24 locus and influence cancer predisposition in a number of different malignancies including colorectal and prostate cancers [115,116,117,118]. Identifying the molecular mechanisms resulting in this outcome remains challenging and requires extensive validation, including analysis of enhancer activity, and with the development of novel technologies to capture chromatin conformations, the interaction of these putative enhancer sequences with the *MYC* promoter. Despite these challenges, several studies have demonstrated a role for a subset of 8q24 SNPs in *MYC* deregulation. For example, the risk allele rs6983267 is significantly associated with the risk of developing colorectal and prostate cancer, with the G variant resulting in increased risk as compared to the reference allele [119,120]. This variant is approximately 335 kb upstream of *MYC*, and was demonstrated to behave as a long-range enhancer that influences *MYC* transcription as a result of altered allele-specific binding of the WNT-pathway transcription factor TCF4 [114,117,121,122]. Another SNP, rs55705857-G, recently found to be associated with increased risk in *IDH1/2*-mutant gliomas, is positioned within a single chromatin topologic domain with *MYC*, and increases the activity of the MYC transcriptional network, as compared to the reference allele [118]. The underlying mechanism and specificity of this risk allele for *IDH*-mutant gliomas is unclear and future work will be needed to delineate this intriguing relationship.

#### 3.2.2. Activation of Enhancer Sequences

Histone acetylation is associated with an open chromatin conformation that is permissive for transcription. In particular, H3K27 acetylation is associated with active promoters and enhancers, and is frequently utilized to identify active enhancers in genome-wide chromatin immunoprecipitation sequencing (ChIPseq) experiments [123]. Deregulation of *MYC* at the endogenous locus can also be achieved through the hyperacetylation and hyperactivation of lineage-specific enhancers that are either absent or less active in normal cells [123]. This has been elegantly demonstrated by several recent publications. For example, a cluster of enhancers located downstream of the mouse *Myc* locus were found to interact with the *Myc* promoter in vivo [124]. This enhancer activity was maintained by the chromatin remodelling SWI/SNF (SWItch/Sucrose Non-Fermentable) complex, which was identified in this study to serve an oncogenic, rather than tumor suppressive function, by maintaining *Myc* expression. Furthermore, the corresponding region in humans, located 1.7 Mb downstream of *MYC*, has previously been identified to undergo focal amplifications in acute myeloid leukemia (AML) and is hyperacetylated in T-cell leukemias and chronic myelogenous leukemia [125,126,127,128,129,130]. Additionally, Herranz et al. identified a recurrently amplified T-cell enhancer in T-cell acute lymphoblastic leukemia, located 1.47 Mb downstream of the *MYC* locus [131]. A technique termed pinpointing enhancer-associated rearrangements by chromatin immunoprecipitation (PEAR-ChIP) has enabled the detection of enhancer rearrangements that result in transcriptional deregulation of oncogenes [132]. This technique was successfully used to identify enhancers deregulating *MYC* expression in B-cells, identifying lineage-specific enhancers located 235 and 535 kb downstream of *MYC*. Moreover, this region was also identified to be a target of recurrent somatic amplifications in plasma cell myeloma, supporting a role for this enhancer in deregulation of *MYC* in B-cell malignancies [133] (summarized in Figure 2b).

Other lineage-specific enhancers have been implicated in malignancies in addition to those of the hematopoietic system. For example, Lin et al., identified a medulloblastoma-specific enhancer 90 kb upstream of *MYC* [64], and similarly, a region 400 to 500 kb upstream of *MYC* serves as a colorectal specific super-enhancer (Figure 2b). Detecting altered enhancer activity in cancer has also been advanced by the integration of non-coding somatic copy number alterations and epigenetic profiling of enhancer marks, facilitating the identification of putative enhancers undergoing focal amplification in multiple different cancer types [110]. In this recent study, non-coding amplifications were identified 450 kb and 800 kb downstream of *MYC*, in lung adenocarcinoma and uterine corpus endometrial carcinoma respectively. These amplifications resulted in the formation of a large enhancer region, as characterized by H3K27 hyperacetylation, resulting in increased *MYC* transcription. Furthermore, an alternative means of enhancer deregulation is through differential methylation of these regulatory domains. DNA methylation is generally associated with transcriptional repression, and hypomethylation of a colorectal specific enhancer was identified as a novel means of activating enhancers, leading to deregulated *MYC* transcription [112].

It is becoming abundantly clear that *MYC* transcriptional regulation is extremely complex. The gene desert surrounding *MYC* houses many lineage-specific regulatory regions that act as modulators of *MYC* expression (also further discussed in Section 5). This is likely due to the cohort of transcriptional regulators that finely-tune cell-type specific *MYC* expression, which is highjacked in cancer to deregulate *MYC*. Further investigation into the discrete mechanisms regulating *MYC* expression in different cell-types has the potential to identify novel means of *MYC* deregulation and inform potential therapeutic vulnerabilities. This concept has already been highlighted by the discovery and use of the small molecule JQ1 as an inhibitor of *MYC* expression in malignancies where *MYC* expression is regulated by large enhancers, such as in MM [128,134].

## 4. MYC Protein Stability

In addition to the complex transcriptional regulation of *MYC*, MYC protein abundance is also tightly regulated. MYC is generally considered to be a short half-life protein and several mechanisms have been identified that contribute to its degradation or stabilization in response to appropriate signaling events. In cancer, these pathways can be altered, resulting in aberrant MYC stabilization, or MYC itself can undergo point mutations that render it unresponsive to specific protein turnover pathways. Findings related to MYC protein stability are summarized in the following section.

### 4.1. MYC Protein Turnover

To date, one pathway has been extensively characterized as modulating MYC protein stability. MYC is first phosphorylated on serine-62 (pS62) by cyclin dependent kinase 1 (CDK1) or extracellular signal-regulated kinase (ERK), among other kinases, followed by phosphorylation of threonine-58 (T58) by glycogen synthase kinase (GSK3) [15,135]. Subsequently, the prolyl isomerase PIN1 isomerizes proline residue 57 from the cis- to trans-conformation, permitting protein phosphatase 2A (PP2A) dephosphorylation of pS62 [136]. Singly phosphorylated T58 is then recognized by the E3 ligase FBXW7, which ubiquitylates MYC and targets it for degradation [44]. Several deubiquitinases have been reported to work in opposition to FBXW7 and stabilize MYC, namely ubiquitin specific protease 28 (USP28), USP36, and USP37 [137,138]. More recently, USP22 has also been reported to regulate MYC stability, however it is unknown whether this is in opposition to turnover mediated by FBXW7 [139]. In accordance with MYC’s oncogenic activity, many of these MYC regulators have been characterized either as oncogenes, such as USP28 [140], or tumor suppressors, for example FBXW7 [141]. Another layer of regulation lies in the three isoforms of FBXW7, and their localization. FBXW7α is found in the nucleus, along with USP28 [137], while FBXW7γ and USP36 are predominantly located in the nucleolus [142]. FBXW7β is found in the cytoplasm and does not interact with MYC [143].

**Figure 3:**
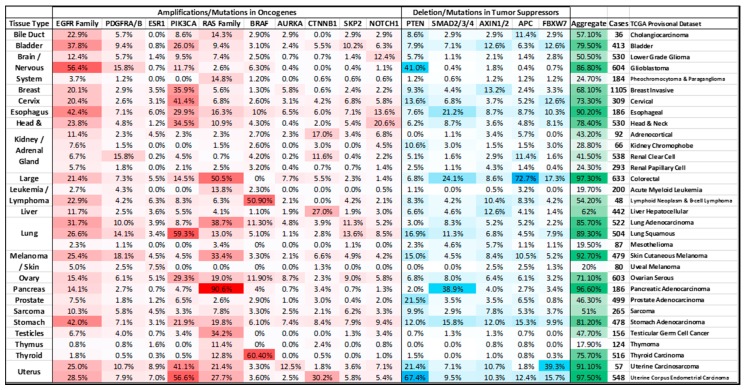
Tabulated summary of genomic alterations (copy number alterations and mutations) in genes known to influence the expression of the *MYC* gene or regulate the MYC protein in 32 cancer types profiled by the TCGA. The data was collected from the TCGA provisional datasets indicated using cBioPortal [34,35]. The “aggregate” column refers to the percentage of tumors of a specific cancer-type that contain genomic alterations in at least one of the genes present in the table.

There are other regulators of MYC stability in addition to the FBXW7 pathway that are altered in cancer. The E3 ligase SKP2 is reported to recognize and ubiquitylate MYC. SKP2 initially enhances MYC’s transcriptional activity, prior to stimulating its protein degradation via the proteasome [144]. This observation was supported by MYC’s recruitment of both SKP2 and components of the proteasome to chromatin, and by the discovery of the MM-1 protein, which interacted with the 26S proteasome and MYC, leading to proteasomal degradation [145,146]. Accordingly, SKP2 is recognized as an oncogene [147] and is amplified in a subset of cancers (summarized in Figure 3). Given that MYC has a short half-life across multiple cell types, it is likely that other FBXW7 and SKP2-independent stability mechanisms exist that have yet to be identified.

### 4.2. MYC Mutations and Protein Stability

While point mutations in c-MYC and N-MYC proteins are relatively rare, a number of studies have identified a subset of functional mutations, primarily targeting MYC Homology Box I (MBI), a highly conserved region in MYC family proteins [73,148,149]. Sequencing studies in BL tumors have identified mutational hotspots in *MYC*, identifying, amongst other point mutations, frequent mutation of the T58 residue [150,151,152,153]. In support of this observation, viral MYC (v-MYC), is known to have potent transforming capacity and also contains a variant of residue 58 (an alanine substitution), resulting in increased protein stability of v-MYC [150,154,155]. This is highly suggestive that MYC stability could be a critical component of transformation, and structure function studies verified that mutation of T58 to alanine yielded an increase in protein stability [88,154]. However, in another study the gain-of-function activity of T58A in a mouse model of B cell lymphomagenesis was attributed, not to an alteration of MYC stability, but to the downregulation of pro-apoptotic BIM expression [156]. Additional hotspot mutations identified in BL, including E39D and T58I, have been found to exhibit elevated transcriptional activity by microarray analyses and demonstrated gain-of-function activities in an in vivo xenograft model [148]. Despite these observations, the T58I mutation observed most frequently in BL was also shown, surprisingly, to have a substantial decrease in transforming capacity in an earlier study [157], bringing into question the pathological relevance of T58 point mutations in human tumors, and potentially implying a context- or tissue-dependent transformative role. Despite this finding, the important role of this pathway in regulating MYC stability is still well-supported.

More recently, Chakraborty et al. described residues elsewhere on MYC that phenocopy T58A [158]. The T244 and P245 residues were identified from a study of hotspot mutations in lymphomas, and the mutation of P245 to alanine (P245A) yielded an increase in half-life similar to what was observed with T58A. Overall, the phosphodegron in the region 244–248 closely resembles that of the 58–62 region, however recognition of this second phosphodegron by GSK3 or FBXW7 has not yet been demonstrated [158]. It remains to be seen whether this is a potential secondary site of FBXW7 regulation or if additional regulators exist for this second phosphodegron. FBXW7 is also commonly mutated in many tumors, including two hotspot residues, arginine 465 (R465) and R479, accounting for ~43% of the observed mutations. The resulting mutant proteins act as dominant negatives of the FBXW7 protein resulting in increased expression of its protein targets, such as MYC to promote tumor growth [141,149].

Analysis of the high-risk neuroblastoma genetic landscape using a combination of next-generation sequencing approaches resulted in the identification of a recurrent mutation in the *MYCN* gene (1.7% of cases, resulting in the P44L missense mutation) [73]. Tumors that harbor the P44L mutation exhibit a very low mutation frequency and were mutually exclusive of *MYCN* amplification. Further analyses will be needed to reveal whether this region is responsible for a gain-of-function phenotype, and if this aberration can be targeted therapeutically in the patients affected. Genomic analysis of 293 basal cell carcinoma (BCC) tumors identified recurrent *MYCN* missense mutations in 30% of these cases, mapping to MBI (P44L, P44S, or P44F) [149]. While it remains to be seen whether this mutation is important for the stability of N-MYC, the authors found that the P44L mutation led to increased auto-ubiquitylation of FBXW7α and represents a unique mechanism for oncogenic N-MYC P44L activity in BCC. The authors also noted a number of additional point mutations within the MBI region of N-MYC, which could impair the interaction with FBXW7α or other regulators of N-MYC stability [146,159].

## 5. Signaling Pathways and MYC Deregulation

Multiple signaling pathways are known to regulate *MYC* gene expression and result in MYC deregulation in the absence of translocation or amplification. Here we discuss evidence of aberrant signaling contributing to *MYC* transcriptional upregulation and/or enhanced MYC protein stability in a variety of tumor types, including chronic myeloid Leukemia (CML), T-cell acute lymphoblastic leukemia (T-ALL), breast, colorectal, and liver cancers.

### 5.1. PI3K/AKT Signaling

**Figure 4:**
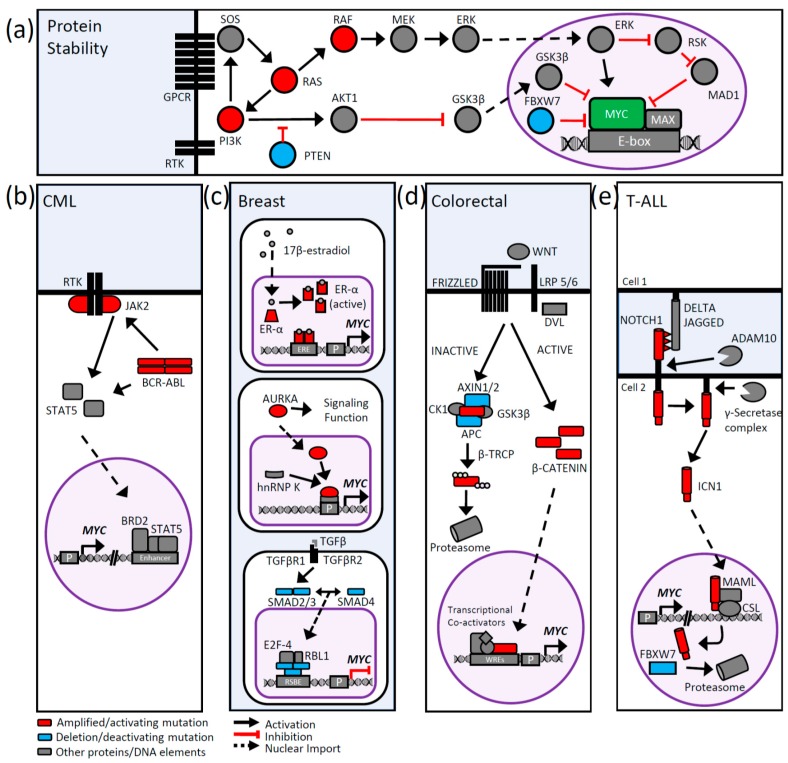
Signaling pathways regulating MYC transcription and MYC protein stability in cancer. (**a**) MYC protein stability can be altered through the RAS/PI3K signaling pathways. (**b**–**e**) *MYC* transcription is deregulated by multiple signaling pathways in many cancer types. Specific signaling pathways are highlighted to demonstrate transcriptional deregulation of the MYC gene in the context of (**b**) chronic myeloid leukemia (CML) (**c**) breast cancer, (**d**) colorectal cancer, and (**e**) T-cell acute lymphoblastic leukemia (T-ALL). Proteins deregulating MYC are highlighted, with red-colored proteins indicating oncogenic alterations (mutation or copy number alteration in greater than 10% of any TCGA cancer dataset) and tumor suppressors shown in blue (mutations and deletions; greater than 10% of any TCGA cancer dataset). Signaling receptors are black and all other proteins are colored grey. RSBE (repressive SMAD binding element), ERE (estrogen response element), WRE (WNT response element), P (Promoter).

The PI3K/AKT pathway is one of the most commonly mutated pathways in cancer [160]. The PI3K protein interacts with receptor tyrosine kinases (RTKs) at the plasma membrane and converts extracellular stimuli into intracellular signals though the phosphorylation of a second messenger molecule phosphatidylinositol biphosphate (PIP2) to PIP3, initiating a signal transduction cascade through the phosphorylation and activation of the serine/threonine kinase AKT [161]. PTEN (phosphatase and tensin homolog) directly antagonizes PI3K activity by dephosphorylating PIP3 back to PIP2 [162,163]. Once activated, AKT acts as an effector kinase, regulating a number of proteins and pathways important for tumorigenesis [164], including glycogen synthase kinase (GSK3) [165]. AKT phosphorylates GSK3 and inactivates the kinase, inhibiting its ability to phosphorylate and negatively regulate MYC protein stability [88] (summarized in Figure 4a). Both *PI3K* and *PTEN* are commonly mutated in many cancers (Figure 3) [160], with activating mutations in the helical domain of the p110α subunit of PI3K (such as E542K and E545K) relieving the inhibitory effects of the p85 regulatory subunit of PI3K [166]. Mutations in the kinase domain of *PI3K*, such as H1047R, have been shown to phosphorylate PIP3 independently of RAS-GTP [166]. Conversely, the *PTEN* gene is commonly lost in many tumors (see Figure 3) [167], with all mutations of these genes leading to hyper-activated AKT [168]. Thus, activation of PI3K/AKT by multiple mechanisms can lead to MYC deregulation by increasing MYC stability.

### 5.2. MAPK Signaling

Ligand binding and activation of RTKs leads to phosphorylation of their C-terminal plasma membrane domains, providing a site of interaction for a number of proteins at the plasma membrane. These proteins then recruit signaling components of the MAPK and PI3K pathways, including the guanine nucleotide exchange factor, son of sevenless (SOS). Localization of SOS at the plasma membrane brings it into the proximity of RAS, allowing SOS to catalyze the nucleotide exchange of GDP to GTP on the RAS family of GTPases (HRAS, KRAS, NRAS), leading to RAS activation [169]. GTP loaded RAS activates the RAF kinases (ARAF, BRAF, CRAF), which in turn activate the MEK kinases (MEK1 and MEK2), leading to activation of extracellular-signal-regulated kinase 1(ERK1) and ERK2 [167]. ERK is one of the major effector proteins of the RAS oncoprotein and is able phosphorylate and regulate a number of protein substrates to propagate the pro-growth and pro-survival signals associated with RAS signaling [170].

One mechanism through which ERK propagates these signals is by the regulating the activity and stability of the MYC protein through phosphorylation of S62 (see also Section 4.1) [88]. Recent findings suggest that ERK may mediate more phosphorylation events on the MYC protein to control its stability, implying a more complex regulatory model [171]. As mentioned, RAS-GTP also binds to and activates the PI3K pathway [172,173,174], eventually leading to the inactivation of GSK3 and stabilization of the MYC protein (Figure 4a) [88]. An additional means of MYC regulation occurs indirectly through MAD1, a member of the MYC/MAD/MAX family of transcription factors which competes with MYC for binding to its obligate binding partner MAX [175,176]. Ribosomal S6 kinase (RSK) is activated by ERK, and in turn phosphorylates MAD1, promoting its ubiquitylation and degradation, and allowing MYC to bind to MAX unhindered, increasing MYC transcriptional output [177].

The RAS family of proteins are commonly mutated in many human cancers, with KRAS being the most commonly mutated family member [160,178]. Mutations of glycine-12 (G12), G13 and glutamine-61 (Q61) are the most commonly observed. These sites reside in the RAS active site and are critical for the hydrolysis of the bound GTP. The inability to hydrolyze GTP to GDP maintains the RAS protein in an active state and perpetuates the signal cascade [178,179,180,181,182]. The RAF family of proteins (primarily BRAF) are also often mutated [160], with the most commonly observed mutations being valine-600 (V600), resulting in a constitutively activated kinase [183,184]. Together, activation of these proteins deregulates MYC through increased protein stability.

### 5.3. BCR-ABL1 Signaling in Chronic Myeloid Leukemia

Chronic myeloid leukemia (CML) is a clonal hematopoietic stem cell disorder dependent on MYC expression and driven by the activated tyrosine kinase activity of the BCR-ABL1 fusion protein [185,186,187]. BCR-ABL1 signaling activates multiple pathways, regulating growth and survival including the RAS and PI3K signal transduction pathways. Additionally, BCR-ABL1 activates the JAK/STAT pathway through interaction [187] and activation [188] of JAK2, as well as directly activating STAT protein (Figure 4b) [189]. Activated JAK2 phosphorylates the STAT5 transcription factor at tyrosine-694, allowing STAT5 to homodimerize, translocate to the nucleus and function as a transcription factor. Once translocated, STAT5 dimers bind to the *MYC*-regulatory 3′ super-enhancer (1.7 Mb downstream of the *MYC* coding region) to regulate *MYC* expression [190,191,192]. Interestingly, STAT5 transcriptional activity is regulated by bromo- and extra-terminal domain (BET) proteins, namely BRD2. Active STAT5 recruits BRD2 to assist in the assembly of the pre-initiation complex and regulate *MYC* transcription, suggesting that bromodomain inhibitors, such as JQ1 derivatives, can be effective in CML [193,194]. In addition to BCR-ABL1 fusions, mutations in the JAK/STAT transduction pathway have been identified to be oncogenic. Gain-of-function JAK2 mutations of valine-617 to phenylalanine (JAK2-V617F) render JAK2 constitutively activated [194,195]. This mutation has been detected across myeloproliferative neoplasms and appears to occur in a mutually exclusive manner with BCR-ABL1 fusions [196,197,198]. Myeloproliferative diseases such as CML, containing activating mutations in the JAK/STAT pathway, should therefore be considered to have deregulated *MYC* expression. Indeed, MYC is essential for BCR-ABL1 mediated transformation [186,187].

### 5.4. Breast Cancer Signaling and MYC

Estrogen-receptor alpha (ERα) is a receptor involved in hormone signaling within breast cells. Activation of ERα, through the binding of 17β-estradiol, allows ERα to homodimerize and interact with DNA directly through estrogen response elements (EREs) or indirectly through interaction with other transcription factors such as JUN, specificity protein 1 (SP1), and nuclear factor kappa B (NF-κB) (Figure 4c). MYC is upregulated by estrogen receptor in ERα positive breast cancer cells, through binding to an enhancer located 67 kb upstream of the transcription start site [199,200,201], and ectopic expression of MYC in cells treated with ERα antagonists can rescue their proliferative defects [202]. Gene expression analysis comparing estrogen-treated to MYC overexpression in breast cancer cells identified that half of estrogen-regulated genes were also MYC-regulated, indicating that high MYC expression levels (as observed in basal/triple-negative subtypes) can compensate for ERα in breast cancers [203,204]. Nonetheless, ERα positive or over-expressed breast cancers associated with luminal A and B tumors [203,205] can transcriptionally target the *MYC* gene and deregulate MYC mRNA expression, independent of *MYC* amplification.

Aurora kinase A (AURKA), which is commonly amplified in a subset of cancers (Figure 3) [206] and has increased mRNA expression in breast cancers [207], has been recently shown to have kinase-independent activities. Through interaction with heterogeneous nuclear ribonucleoprotein K (hnRNP K), a protein with nucleic acid binding properties, AURKA binds a promoter element approximately 100–150 bp upstream of the MYC P1 promotor, inducing a shift in the ratio of P1 vs. the shorter P2 transcript of MYC. In this manner, AURKA can act as a transcriptional regulator to induce *MYC* expression in human breast cancer cell lines (Figure 4c) [207,208].

Repression of MYC through transforming growth factor β (TGF-β) signaling is an important method of negatively regulating *MYC* expression in some breast cancers. Upon binding of the TGF-β ligand to the TGF-β receptor, SMAD2/3 becomes phosphorylated and active, leading to the formation of a repressive complex in the proximal promotor region of *MYC* (Figure 4c) [209,210]. Loss of this negative regulatory pathway and subsequent decreased nuclear SMAD3 has been shown to correlate with tumor aggressiveness [211,212]. In addition, this pathway can be deregulated in the context of active RAS signaling, through JNK-mediated SMAD3 signaling [213]. While *MYC* amplification is associated with basal breast cancer (see Section 2.1, Figure 1a,b), other subtypes of breast cancer possess the signaling pathways required to deregulate *MYC* expression independent of this gross genetic abnormality.

### 5.5. WNT Signaling and MYC

Aberrant activation of the canonical WNT-β-catenin signaling pathway is estimated to occur in more than 90% of colorectal cancers [214]. In the absence of WNT ligand, cytoplasmic β-catenin is continually degraded intracellularly via β-catenin phosphorylation and a ‘destruction’ complex containing the axis inhibition proteins 1 and/or 2 (AXIN1/2), adenomatous polyposis coli (APC), casein kinase 1 (CK1), and glycogen synthase kinase three (GSK3) (Figure 4d) [129,215]. While APC and the AXINs act as a scaffold to position β-catenin, the latter enzymes phosphorylate β-catenin marking it for ubiquitylation by the β-transducin-repeat-containing protein (β-TRCP) and subsequent degradation via the proteasome. In the absence of nuclear β-catenin, T-cell factor/Lymphoid enhancer factor (TCF/Lef) family of sequence-specific transcription factors and transducing-like enhancer protein (TLE/Groucho) form a repressive complex, which recruits histone deacetylases (HDACs) to WNT responsive elements (WREs) and prevents the transcription of WNT pathway target genes, including *MYC* [129,215]. When the ligand is present, Disheveled (DVL) proteins associate with the Frizzled-LRP5/6 receptors and result in the inactivation of the intracellular destruction complex through segregation of the AXINs. This allows stabilized β-catenin to translocate to the nucleus where it forms active transcriptional complexes by displacing the repressive complexes off of TCF/Lef bound WREs and recruiting other activating co-factors, resulting in the transcriptional activation of pathway target genes [129,215]. Of the many target genes transcribed by this pathway, *MYC* is one of the best characterized [39,122,216,217]. WREs have been identified both upstream and downstream of the MYC gene boundary, and form a loop that accompanies *MYC* transactivation [130]. In colorectal cancers, the predominantly mutated component of this signaling pathway is the tumor suppressor gene *APC* with 85% occurring in a mutational “hotspot” region of the gene (see Figure 3).

In addition to *APC* mutations in colorectal cancers, WNT signaling can also be aberrantly activated via mutations that stabilize β-catenin or impaired AXIN1/2 function, although these are less ubiquitously found [217,218,219,220]. For example, there is evidence of MYC deregulation through altered WNT signaling in pediatric hepatoblastoma (HB) and hepatocellular carcinoma (HCC) [221,222,223]. Individuals with an inherited familial *APC* gene mutation have and 750–7500 times higher risk for the development of HB as young children [224], and greater than 80% of HBs have a mutation in β-catenin, APC, or AXIN [225]. Furthermore, in an animal model of HB, Myc was found to be required for sustained tumor growth [226]. *CTNNB1* mutations are also observed in HCC, although with a lower frequency of 15.9% of cases [227,228].

### 5.6. NOTCH Signaling and MYC

One of the hallmarks of T-cell acute lymphoblastic leukemia (T-ALL) is its very high proliferative rate due to the activation of oncogenic pathways closely linked to the mechanisms responsible for driving proliferation and cell growth in early T-cell progenitors [229]. One of these key oncogenic pathways that has been extensively examined for its contribution to T-ALL is the NOTCH signaling pathway [230]. In general, this signaling pathway requires one of four mature NOTCH receptors to bind its candidate Delta/Serrate/Lag-2 (DSL) family of ligands at the cell surface of neighboring cells (Figure 4e). Once this interaction occurs, this prompts the cleavage of the extracellular domains of the receptor by ADAM (a disintegrin and metalloproteinase) metalloproteases which subsequently facilitate another proteolytic cleavage within the transmembrane region of the receptor by the γ-secretase complex. Once the receptor is released from the membrane, the cytoplasmic intracellular portion of NOTCH1 (ICN1) is able to translocate into the nucleus where it directly participates in a core transcriptional complex with several factors including the DNA-binding protein CBF/RBPJκ/Su(H)/Lag1 (CSL) and Mastermind-like transcriptional co-activators (MAML) [230]. This complex interacts with a long-range *MYC* enhancer, located 1.47 Mb downstream of *MYC* [128]. In T-ALL, NOTCH signaling occurs through NOTCH1, which was first implicated in T-ALL pathogenesis as part of the chromosome translocation t(7;9)(q34;q34.3), accounting for 1% of T-ALLs seen in the clinic [231]. However, this rare chromosomal rearrangement led to the later discovery of activating NOTCH1 mutations found to be present in over 65% of T-ALLs [232]. These mutations allow for sustained, high-level expression of NOTCH pathway target genes, of which MYC features prominently. Additionally, about 20% of all T-ALLs contain mutations in *FBXW7*, which can deregulate MYC and NOTCH1 via inhibition of protein turnover [43,44,233,234,235].

## 6. Conclusions

The reports described here, as well as many additional studies, have highlighted that MYC is one of the most frequently deregulated oncogenes in cancer. MYC is clearly the target of many different forms of deregulation in cancer, including gene amplification, and transcriptional induction through altered cellular signaling (summarized in Figure 3) or enhancer activation. This overview highlights only a select few of the pathways contributing to MYC deregulation, and due to the central role of MYC in the proliferation and transformation in many diverse tissue types, it is likely that there are yet undiscovered means of MYC deregulation in cancer; it is therefore extremely difficult to definitively state how often MYC truly is deregulated. Going forward, quantification of MYC protein levels through tumor proteomics [236,237,238] and a more precise definition of MYC activation and/or the MYC transcriptional program may facilitate a better understanding of MYC deregulation in oncogenesis. Moreover, MYC is known to be highly modified at the post-translational level, with many phosphorylation [14,88,136,239,240,241,242], SUMOylation [243,244,245], acetylation [246,247], and ubiquitylation [43] sites identified [248]. In addition to canonical measures of MYC deregulation through genetic or epigenetic alterations, post-translational modifications (PTMs) can also modify MYC activity. Future studies delineating the relative importance of these PTMs and the writers and erasers of these marks will be informative for the future of MYC-directed therapeutics, with the potential to modulate MYC activity at the protein level.

Despite being essential for normal cellular proliferation [5,6], there is evidence that targeting MYC in cancer is a viable option [249]. A proof-of-concept study for MYC inhibition was performed using a dominant negative protein inhibitor of MYC, termed Omomyc [250]. Metronomic expression of systemic Omomyc in a model of RAS-induced lung adenocarcinoma resulted in rapid and sustained regression of lung tumors in vivo, with minimal toxicity to normal tissues [251]. Moreover, as MYC is a driver of many diverse cancer types, directly inhibiting MYC in cancer has the potential to have broad therapeutic impact [252]. Delineating how MYC is deregulated in cancer will be important to inform the optimal strategies to use for the development of anti-MYC therapeutics in the age of personalized cancer medicine. This concept has been exemplified by the recent development of the BET bromodomain inhibitor JQ1, a drug that has shown efficacy in preclinical models of MM [134]. Further understanding of the regulatory mechanisms controlling MYC has the potential to unveil several classes of novel anti-MYC, targeting a wide-variety of malignancies that exhibit activation of the MYC transcriptional network.
